# Assessment of Various Factors for Feasibility of Fixed Cantilever Bridge: A Review Study

**DOI:** 10.5402/2012/259891

**Published:** 2012-03-01

**Authors:** Ashu Sharma, G. R. Rahul, Soorya T. Poduval, Karunakar Shetty

**Affiliations:** Department of Prosthodontics, Bangalore Institute of Dental Sciences and Research Center, 5/3 Hosur Main Road, Oppasite Lakkasandra Bus Stop, Wilson Garden, Bangalore 560027, India

## Abstract

Cantilever fixed partial dentures are defined as having one or more abutments at one end of the prosthesis while the other end is unsupported. Much controversy without documentary evidence has surrounded this prosthesis. Despite negative arguments, the cantilever prosthesis has been used extensively by the clinicians. If used nonjudiciously without following proper guidelines these might lead to some complications. Although complications may be an indication that clinical failure has occurred, this is not typically the case. It is also possible that complications may reflect substandard care. Apart from the substandard care, the unique arrangement of the abutments and pontic also accounts for the prime disadvantage: the creation of a class I lever system. When the cantilevered pontic is placed under occlusal function, forces are placed on the abutments. There are various criteria and factors necessary for a successful cantilever fixed partial denture (FPD). The purpose of this paper is to discuss briefly various factors involved in the planning of a cantilever fixed partial denture.

## 1. Introduction

Because of the requests for fixed partial prostheses by patients and because of the extensive restorative procedures used in complete oral rehabilitation, many dentists have been using these partial dentures with free-end pontics. Dentists have used this type of restoration for several years with more than moderate success. However, during this period, the number of failures observed has been too high to be considered the result of accident. Too many roots and crowns fractured of the abutment teeth adjoining the free-end pontic. Also, some of the gold crowns that covered these terminal abutment teeth loosened without the crowns covering the remaining portion of the splint loosening. This was not always detected until caries had caused acute dental pain which involved the pulp and destroyed the crown of the tooth next to the cantilevered pontic. Therefore, it became necessary to probe deeper into the problem [1].

Every dentist realizes that important correlations exist between biology and mechanics in treating patients with either fixed or removable partial dentures. A distribution of stress within physiologic limitations of supporting structures in both types of restorations has been a vital consideration of dentists for many years. It is not uncommon to be confronted with oral situations in which treatment with either a distal extension removable partial denture or a posterior, cantilever type of fixed partial denture is considered. 

**Figure 1:**
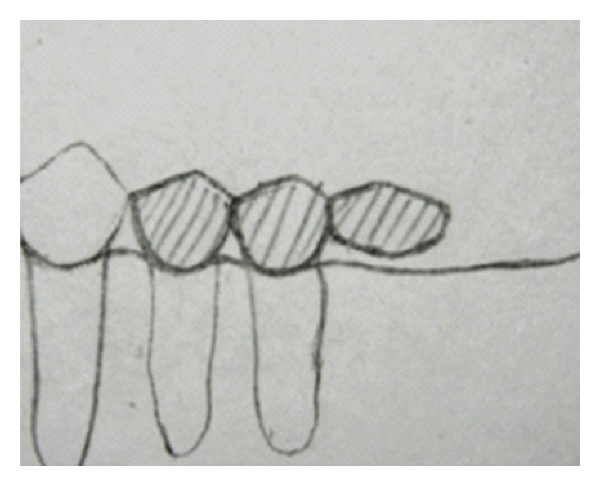
Posterior cantilever bride [2].

**Figure 2:**
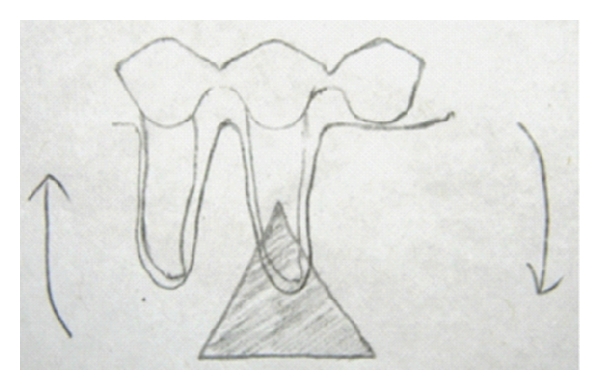
Creation of class I lever system is primary disadvantage of cantilever fixed partial denture [3].

The cantilever fixed partial denture is defined as a fixed restoration which has one or more abutments at one end while the other end is unsupported. Varied and extensive clinical experience is certainly of great value in treatment planning and weighing the relative merits of a particular type of restoration for a given clinical situation. However, we also learn from the experiences of others—our peers and those who have preceded us. Yet, there is a dearth of information concerning the posterior, cantilever type of fixed partial dentures in dental periodical literature and in current textbooks due to which there has been a lot of cantilever FPD cases failures [2] (Figures 1 and 2).

## 2. Search Strategy

A PubMed search of English literature was conducted up to January 2010 using the terms: Free-end fixed partial denture, Distal extension fixed partial denture, and Cantilever fixed partial denture. Additionally, the bibliographies of 5 previous reviews, their cross-references as well as articles published in Endodontic Dentistry, Journal of Clinical Periodontology, Journal of Prosthodontics, Journal of Oral Rehabilitation, British Dental Journal, International Dental Journal, Journal of prosthetic dentistry, and Swedish Dental Journal were manually searched.

## 3. Prerequisites for Successful Cantilever Fixed Partial Dentures

Ewing lists the following factors when making a physiologic appraisal before employing the cantilever principle: (1) good periodontal attachment (covering maximum rot surface), (2) good alveolar support, (3) favorable root length, shape, and crown length, (4) favorable arch-to-arch relationship, (5) favorable tooth-to-tooth relationship. The same factors certainly enter into diagnosis and treatment planning with removable partial dentures. 

**Figure 3:**
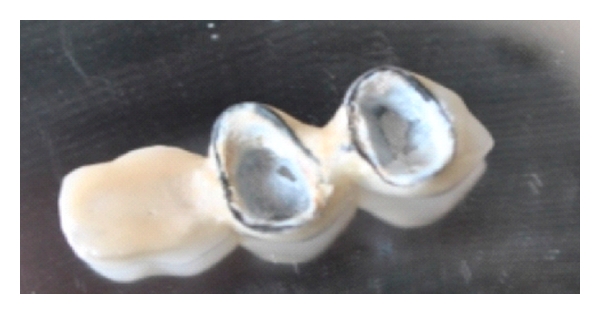
Failed posterior cantilever bridge due to fractured premolar abutment.

**Figure 4:**
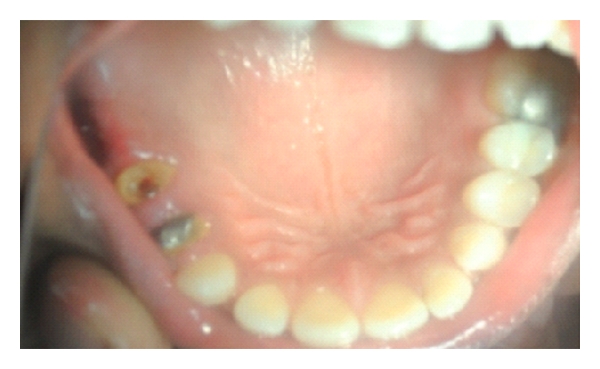
Fractured 2nd premolar (abutment next to pontic).

Ante proposed, in selecting the number of abutments for a fixed restoration, that “the total periodontal membrane area of the abutment teeth should equal or exceed that of the teeth to be replaced.” This formula is accepted today as a meaningful guide by the authors of many current textbooks on fixed partial dentures.  Table 1 shows the root surface areas of periodontal membrane attachments of average normal teeth as computed by Tylman. Applying Ante's general rule, it can be seen that the total area of periodontal membrane attachment of the lower premolars on one side is 265 sq. mm. These two teeth should then adequately support a canine pontic but not a first molar pontic. On the other hand, the total periodontal area of the second premolar and first molar is 487 sq. mm. and should support a second molar cantilevered pontic. However, Tylman states that “great caution and reserve are essential whenever an attempt is made to interpret biological phenomena entirely by mathematical computation.” Here again, varied and extensive clinical experience becomes a most important factor in treatment planning (Figures 3 and 4).

### 3.1. Anterior Cantilever Fixed Partial Dentures

Cantilevered fixed partial dentures can be more successful in anterior quadrant than posterior because the forces are less in anterior region than posterior one. The cantilevered FPD requires at least two abutment teeth. The only documented exception permitting a single abutment is the replacement of a maxillary lateral incisor with the canine as an abutment [4]. This anterior cantilever can be the ideal indication for cantilever fixed partial denture. Anterior cantilever fixed prostheses are indicated for open bite conditions or where a normal degree of vertical and horizontal overlap is present. 

However, anterior cantilever fixed partial dentures are not indicated where excessive vertical overlap is present, because the anterior teeth are subject to excessive loading in protrusive and lateral excursions [5]. Cantilever prostheses are not indicated for patients with Class III malocclusions who exhibit excessive wear patterns on anterior teeth [5].

### 3.2. Stress Distribution, Masticatory Forces, and Their Action upon Prosthetic Appliances

Forces applied to the cantilevered pontic are resisted through rotational and tilting movements by the abutment teeth rather than those along the long axes [2]. To preserve the integrity of the supporting periodontium and prevent material failure, it is crucial to understand the nature of each component of the prosthesis. 

**Figure 5:**
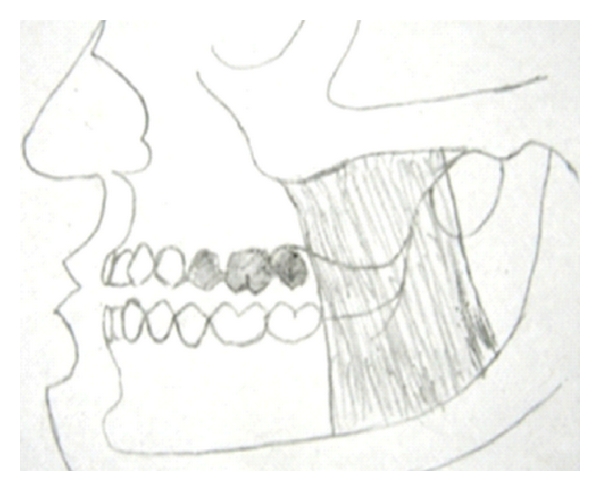
Pontics extending into posterior regions of dental arch are subject to increased loads because strong masticatory muscles are located closer to the posterior pontics and angle of mandible.

Single cantilevered pontics with at least two abutments are recommended [1], although this may vary depending on the existing clinical conditions and the location of the pontic in the dental arch. The muscles of mastication cause the strongest forces to be applied in the posterior regions of the arch. When a cantilevered pontic is placed posteriorly, additional abutments may be required to withstand the forces [3] (Figure 5).

Henderson et al. [2] used a practical model and a laboratory model of a three-abutment posterior FPD with strain gauges. In both models, forces transmitted to the abutments through the cantilevered pontics were resisted by rotational and tilting movements of the abutments but not parallel to the vertical axis of the abutment roots. More than 50% of the force applied to the cantilever pontic was absorbed by the abutment nearest the cantilever pontic, but the addition of abutment teeth lessened the force on the distal abutment. It was concluded that the abutment nearest the pontic of a cantilever type of fixed partial denture will assume more than 50 per cent of the load placed against the pontic. However, a three-abutment cantilever restoration will materially lessen the “combined total resultant” forces to the distal abutment compared to a two-abutment cantilever restoration.

### 3.3. Cross-Arch Cantilevered FPDs (Unilateral and Bilateral)

The cross-arch unilateral two-unit cantilever FPD was examined by Lundgren and Laurell [6]. Strain gauge transducers were inserted into prosthetic restorations to register occlusal forces during light tooth-tapping, chewing, swallowing, and maximal biting. They demonstrated that, despite the activity, the distal cantilevered unit was subjected to comparatively less stress than the contralateral posterior abutment with equal or smaller than local anterior forces. The diminished forces on the cantilever units are attributed to a deflection of the cantilever units and not to intrusion of the adjoining abutments.

In a comparative study of patients restored with cross-arch FPDs with bilateral terminal abutments, an average of 26% of the voluntary muscular capacity was activated during chewing compared with 37% in the bilateral terminal abutment group [7]. The differences were explained by the unilateral lack of terminal abutments causing lateral bending forces that activate peripheral inhibitory feedback reactions from the periodontal and/or TMJ mechanoreceptors.

### 3.4. Role of Occlusion

Longitudinal studies [8, 9] have confirmed that dentition can be rehabilitated by use of FPDs with cantilever pontic on specific, isolated abutments that are periodontally compromised. Stable FPDs were successful despite individual hypermobile abutment teeth. Prolonged stability was achieved by periodontal treatment and the development of a stable, nontraumatizing occlusion. Balancing contacts were established to prevent migration, tilting, and increasing mobility when there was a possibility of FPD mobility during mandibular movements.

The masticatory forces are decreased with periodontally compromised teeth in dentitions with cross-arch unilateral posterior two-unit cantilever FPDs. The quadrants with the cantilevers were never designated as the preferred chewing side [10]. Thus, if the occlusion is stable and the cantilever free from premature contacts, the cantilever would be only inadvertently subjected to large forces.

A 10 year follow-up study was done by Hochman et al. [3] on anterior and posterior cantilever fixed partial dentures, in which two kinds of abutment crowns were used in which two kinds of abutment crowns were used. In the earlier years gold veneer crowns were used. In the later years, metal-ceramic restorations were used. Periodontal status was periodically evaluated and the occlusion was adjusted as necessary. Special instructions for cleaning interproximal surfaces and under the pontics were given to the patients. Group function occlusion was preferred, for fabrication of FPD, in lateral and protrusive excursions for the cantilever unless definite canine protected occlusion was present. The cantilever pontics in posterior regions were adjusted to receive light occlusal contact.

Patients were periodically observed for 10 years and observations were recorded. None of the patients had subjective (esthetic or functional) complaints. No noticeable changes were observed in the abutment teeth compared with the homologous teeth (same set of teeth) on the opposite side of the arch and with original radiographs. Minor occlusal adjustment was sometimes necessary to prevent occlusal trauma from uneven attrition [5].

### 3.5. Type of Dentition Opposing the Planned Cantilever Prosthesis

Antonoff [11] stated that cantilever FPDs were more frequently indicated when reduced stress was inherent, as with a complete denture in the opposing dentition. However, Randow et al. [12] reported no major clinical significances between technical failures of cantilevered FPDs and the type of opposing dentitions. They suggested that a well-supported, stable complete denture could also generate high functional loading.

#### 3.5.1. Proper Design of the Cantilevered Pontic

In order to decrease the load from free-end pontics, the pontics have been made with facings but without complete occlusal surfaces. This does decrease the vertical and horizontal pressures on the pontics. Care should be taken to make certain that the interocclusal relationship is such that the opposing teeth will not “overerupt” because of lack of occlusal contact. The excessive eruption of teeth creates a condition which may prove to be equally as unsatisfactory as the excess occlusal force that one is trying to avoid [1].

Esthetic results can often be attained with cantilever prostheses where reduced mesiodistal space is available. The pontic should not exert pressure on the gingival tissue. Accessory supports from rests on adjacent teeth are contraindicated because of the possibility of caries under the rest and the difficulty of maintaining proper hygiene [7]. 

#### 3.5.2. Mechanical Features of FPD

**Figure 6:**
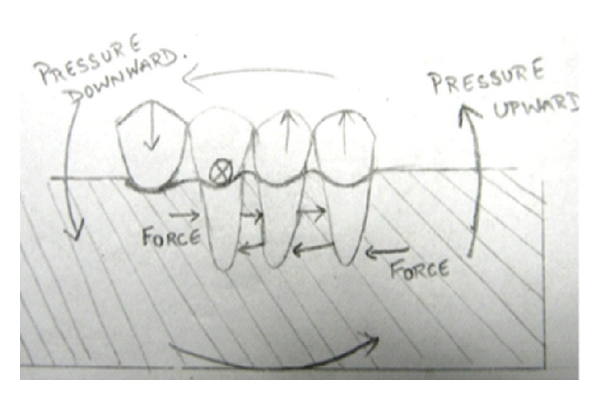
Forces exerted on the most posterior free-end pontic cause the greatest displacing pressure on the most anterior abutment casting. The cement must be strong to oppose severe compressive and tensile pressure without disintegrating.

**Figure 7:**
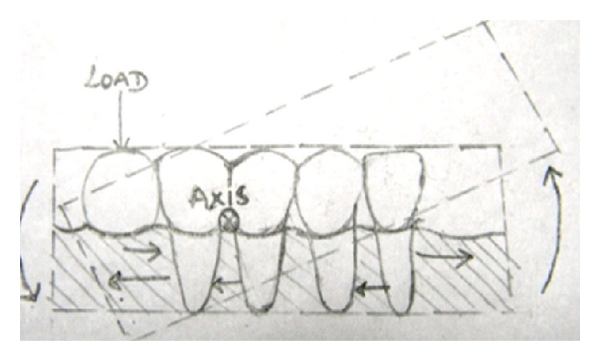
The load directed downward on the free end pontic causes the rectangle (dotted line) to rotate on an axis shown in the diagram. This demonstrates the compressive and tensile effects and the need for strong cements and strong soldered joints as well as strong metal for castings.

The greatest dislodging forces arc met in the abutment tooth farthest from the free-end pontic. If the forces which tend to rotate the prosthesis occur around the axis of rotation in the sagittal plane, which is in or close to the abutment tooth adjoining the free-end pontic, it is not difficult to understand that the forces tending to lift the cast crown from the abutment farthest from the free-end pontic are greatest. At the clinical level, this means that the cement should be strongest where the forces of compression and distension are greatest and, also, that the metal should be strongest over these abutment teeth [1]. The maximal strength of most luting cements is compressive, the minimal strength is tensile, and the shear strength has an interval value. Apically directed forces on the cantilever direct tensile forces to the cement of the retainer farthest from the cantilever [6] (Figures 6 and 7).

#### 3.5.3. Biological Features of Cantilever FPD

 Axially directed masticatory forces are more consistently influenced by the periodontal support with cross-arch extension FPD with unilateral cantilevers [10]. The less the periodontal ligament area is, the smaller the occlusal force exerted, suggesting that the mastication of dentitions with unilateral posterior two-unit cantilevers is modulated more by the mechanoreceptors of the periodontal membrane than by dentitions with bilateral, terminal abutments. Excessive bending forces from the cantilevers can alter the feedback control mechanism from the periodontal mechanoreceptors, magnifying neuromuscular sensitivity. However, the periodontal tissues do not affect the local forces on the distal unit of the cantilever because of the deflection of the cantilever [6].

The importance of the mechanoreceptor mechanism of the periodontal membrane was emphasized by Randow and Glantz [13]. They discovered that there was a definite difference in the biomechanic reactions on cantilever loading between vital and nonvital teeth. The tolerable loading levels in nonvital teeth were twice this of vital teeth. Nevertheless, the vital and nonvital teeth had the same level of tolerable loading when the abutments were anesthetized. They concluded that the vital teeth with optimal bone support had a more efficient form of mechanoreceptor function at lower degrees of bending than nonvital teeth. This elevated response may also explain the greater mechanical failure associated with endodontically treated abutment teeth [3]. Landolt and Lang [14] confirmed that RCT teeth showed a higher frequency of root fracture. Karlsson [15] remarked that the combination of a cantilever extension and an RCT terminal abutment was predisposed to failure.

A Medline and an extensive hand search were performed on English-language publications, covering the last 50 years,s focusing specially on publications that contained clinical data regarding success/failure/complications. There are 12 clinical studies that report the total number of posts and cores evaluated and the total number of complications encountered. There were 279 complications found among the 2784 posts and cores in the 12 studies, producing a mean complications incidence of 10%. The following 4 complications were evaluated in many of these studies: post loosening, root fracture, caries, and periodontal disease. The frequency of occurrence of post and core complications was post loosening (5%), root fracture (3%), caries (2%), and periodontal disease (2%) [16–26].

The causes of failure can be divided into biologic (82%) and technical (18%) failures. Loss of retention with or without caries was the most frequent biologic complication. Technical failures included abutment fracture, prosthesis fracture, and fracture of the cantilevered extension.

#### 3.5.4. Cantilever FPD versus Removable Partial Denture (RPD)

There may be indications in elderly patients to replace lost molar or premolar teeth, to provide satisfactory masticatory function, and to maintain occlusal as well as neuromuscular stability. In patients wearing a complete maxillary denture, prosthetic treatment in the mandible with bridgework or a removable partial denture (RPD) may also be indicated in order to increase occlusal support and stability of the maxillary denture [27].

Clinical evaluation of patients 8–10 years after treatment with RPDs or fixed bridgework has demonstrated that such treatment will not contribute to dental caries or periodontal breakdown provided that the patients' oral hygiene is well controlled [28–31]. However, RPDs, particularly in the mandible, may cause difficulties of adaptation [32].

It has previously been reported that simple distally extending cantilever bridges are a favorable alternative to RPDs in patients with anterior teeth and one or two premolar teeth left in the mandible since a pronounced improvement of chewing function and stability of the maxillary full denture was observed even by patients who previously had been well-adapted wearers of RPDs [33].

Budtz-Jorgensen and Isidor [27] in their study compared prosthetic, functional, and occlusal conditions in twenty-seven patients treated with distally extending cantilever bridges and twenty-six patients treated with removable partial dentures (RPD) in the mandible. All patients had a complete upper denture. Mean age of the patients in both groups was about 69 years. The patients were under a supervised oral hygiene care throughout the 2-year study period. During the study period signs and symptoms of mandibular dysfunction became significantly aggravated in the RPD group, *P*< 0–05. A balanced occlusion in the muscular contact position was observed in 90% of the patients in the bridge group and in 76% of the RPD wearers. During the study period the need for dental or prosthetic treatment was negligible in the patients treated with bridges. In the RPD group, twenty-two teeth were restored with fillings due to caries and in eight patients major adjustments of the sublingual bar were necessary due to irritation of the oral mucosa. This study has shown that treatment with distally extending cantilever bridges in the mandible is a favorable alternative to treatment with removable partial dentures in elderly patients with a reduced dentition.

Clinical findings in a 5-year longitudinal study done by Budtz-Jørgensen and Isidor [34] are shown in Table 2. This study has confirmed previous observations that treatment with distally extending cantilevered FPDs is a favorable alternative to treatment with RPDs in elderly patients. Furthermore, occlusal stability and denture stability deteriorated more often in the RPD group than in the FPD group.

#### 3.5.5. Concept of Increased Interabutment Distance for Cantilever Fixed Partial Denture (EC-FPD)

**Figure 8:**
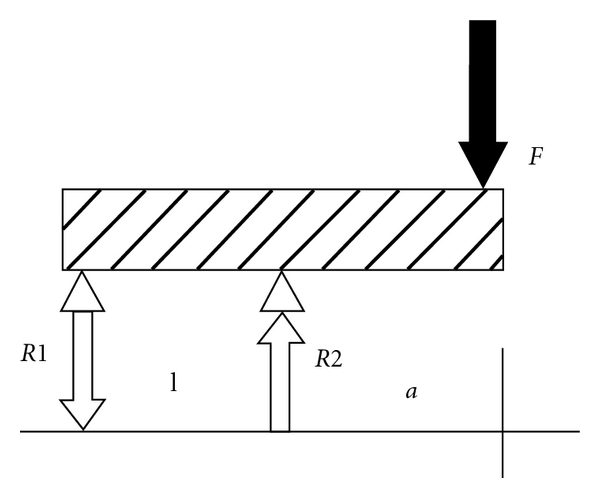
Cantilever FPD retained by 2 adjacent abutments. *F*: applied force; *R1* and *R2*: reactive forces; *a*: cantilever length; *l*: interabutment span [37].

**Figure 9:**
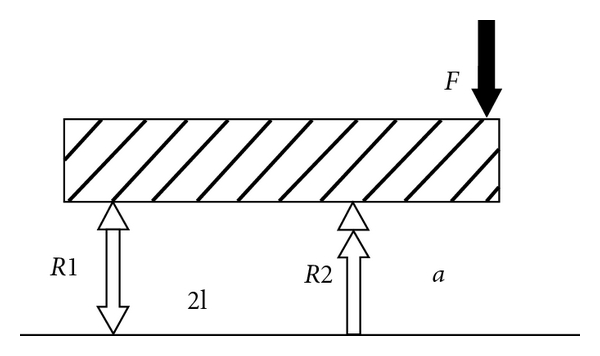
Cantilever FPD with enlarged interabutment span. R1 and R2, Reactive forces; a, cantilever length; 2l as interabutment length.

An analysis was conducted on a cantilever FPD supported by 2 abutments [35].  Figure 8, simulates a cantilever FPD (C-FPD) supported by two abutments with a concentrated load “F” acting vertically on its edge. It was suggested that increasing the span between abutments may reduce the reactive forces and stresses in the 2 distal abutments.  Figure 9 simulates a cantilever FPD supported by two abutments but with an enlarged interabutment span (EC-FPD) compared to Figure 8. Theoretically, increasing the interabutment span from l to 2l could possibly reduce the reactive forces by 25%, from 2 to 1.5 F for C-FPD and EC-FPD, respectively. Similarly, it can be seen that R1 could possibly be reduced by 50%, from -*F* to -*F*/2 for C-FPD and EC-FPD, respectively. According to the analysis, in the situation described, increasing the span between the proximal abutments that support a cantilever FPD could reduce the reactive forces in the abutments between 25% and 50%.

This analysis was established on accepted engineering principles of beams [35]. This is a simulative mathematical method that is based on statically determinate force systems in equilibrium [36, 37] It provides theoretical qualitative information about shear forces, bending moments, and deflections of beams [37]. This concept has been successfully used by Lewinstein et al. [37] in treatment of patients with shortened dental arch.

## 4. Conclusion

It can be concluded from this paper that the current, optimal treatment for replacing missing  teeth is an FPD secured at both ends. The cantilever is considered a compromise but is preferred to the RPD, especially for unilateral edentulous dentitions. There is consensus that an increase in abutment teeth with a reduction in the number and size of cantilevered pontics is essential. Although abutments should have suitable periodontal support, investigators have demonstrated that extensive cross-arch FPDs with cantilevers can be inserted with a minimal periodontal ligament if the occlusion is stable and harmonious. The deflective capacity of the cantilever with the stimulation of the mechanoreceptors in the periodontium reduces the stress on the restoration aiding the compromised periodontal ligament.

Technical failures are more common when nonvital teeth are abutments, because deterioration of tooth structure can be insidious. More occlusal force can also be inadvertently extended to nonvital teeth because their pain threshold is more tolerant. 

Geriatric patients prefer the comfort of a cantilever FPD to an RPD, and less maintenance is required at subsequent appointments. With the rapid advancement of osseointegrated implants, the cantilever FPDs may be used sparingly.

## Figures and Tables

**Table 1:** The root surface areas of periodontal membrane attachments of average normal teeth [2].

Maxillary	Periodontal membrane attachment (sq. mm.)	Mandibular	Periodontal membrane attachment (sq. mm.)
Central incisor	139	Central incisor	103
Lateral incisor	112	Lateral incisor	124
Canine	204	Canine	159
First premolar	149	First premolar	130
Second premolar	140	Second premolar	135
First molar	333	First molar	352
Second molar	272	Second molar	282
Third molar	197	Third molar	190

**Table 2:** Clinical findings in a 5-year longitudinal study done by Budtz-Jørgensen and Isidor [34].

Findings	FPDs with distal cantilever(27 patients)	RPDs(26 patients)
Dental caries	10	57
Endodontic complications	2	5
Tooth fractures	2	5
Denture stomatitis	15	17
Denture ulcer	4	7
Irritation from sublingual bar	—	12
Prosthesis failures	8	—
Denture failures	1	10
Clasp fractures	—	4
